# Autoimmune Hepatitis With Concomitant Pernicious Anemia: A Rare Association

**DOI:** 10.7759/cureus.15045

**Published:** 2021-05-15

**Authors:** Amir Riaz, Sikandar Khan, Rafael Miret, Pablo Bejarano, Asad Ur Rahman

**Affiliations:** 1 Internal Medicine, Cleveland Clinic Florida, Weston, USA; 2 Pathology, Cleveland Clinic Florida, Weston, USA; 3 Gastroenterology, Cleveland Clinic Florida, Weston, USA

**Keywords:** pernicious anemia, autoimmune hepatitis, parietal cell antibody, intrinsic factor blocking antibody

## Abstract

The co-occurrence of autoimmune hepatitis (AIH) and pernicious anemia (PA) is extremely rare. We present a case of a 70-year-old woman with AIH who presented for the evaluation of poor appetite and weight loss. Laboratory studies were significant for microcytic anemia, B12, and iron deficiency. Esophagogastroduodenoscopy showed diffuse gastric mucosal atrophy, and the pathology from the body of the stomach showed chronic gastritis. Additional testing was positive for parietal cell antibody and intrinsic factor blocking antibody, confirming the diagnosis of PA. To the best of our knowledge, there is only one documented case of AIH associated with PA.

## Introduction

The co-existence of autoimmune diseases is well recognized and commonly observed. Approximately 25% of patients with autoimmune disease tend to develop additional autoimmune diseases [1]. Similarly, autoimmune hepatitis (AIH) has been seen with many different autoimmune diseases. Some of the common associations include autoimmune thyroiditis, vitiligo, rheumatoid arthritis, celiac disease, systemic lupus erythematosus, type 1 diabetes, multiple sclerosis, polymyalgia rheumatica, and urticaria [2]. However, the co-existence of AIH and PA is extremely rare. We present a case of AIH associated with pernicious anemia (PA).

This case was previously presented as a poster at the American College of Gastroenterology (ACG) 2020 Virtual Meeting on October 23, 2020.

## Case presentation

A 70-year-old woman of Iranian origin presented to the gastroenterology clinic complaining of poor appetite and a weight loss of 8 lb over the last two months. Her past medical history was notable for type 1 AIH, hypothyroidism, and Takotsubo cardiomyopathy. In addition to her poor appetite and weight loss, she reported mild hoarseness of voice and depression in the setting of her brother recently being diagnosed with bladder cancer. She attributed her poor appetite and depression to the death of her brother. She denied any changes in bowel habits, hematochezia, melena, hematemesis, heartburn, dysphagia, or odynophagia. In regard to her AIH, she was currently in clinical and biochemical remission on a stable dose of azathioprine. Recent laboratory studies demonstrated normal liver function tests, thyroid-stimulating hormone level, and IgG (immunoglobulin G) levels. Her family history was negative for gastrointestinal cancer. She denied alcohol use, cigarette smoking, or illicit drug use. Complete blood count revealed a mean corpuscular volume (MCV) of 87.3 fL (reference range: 80-100 fL), iron of 21 mcg/dL (reference range: 45-160 mcg/dL), and B12 of 261 pg/mL (reference range: 200-1,100 pg/mL).

**Figure 1:**
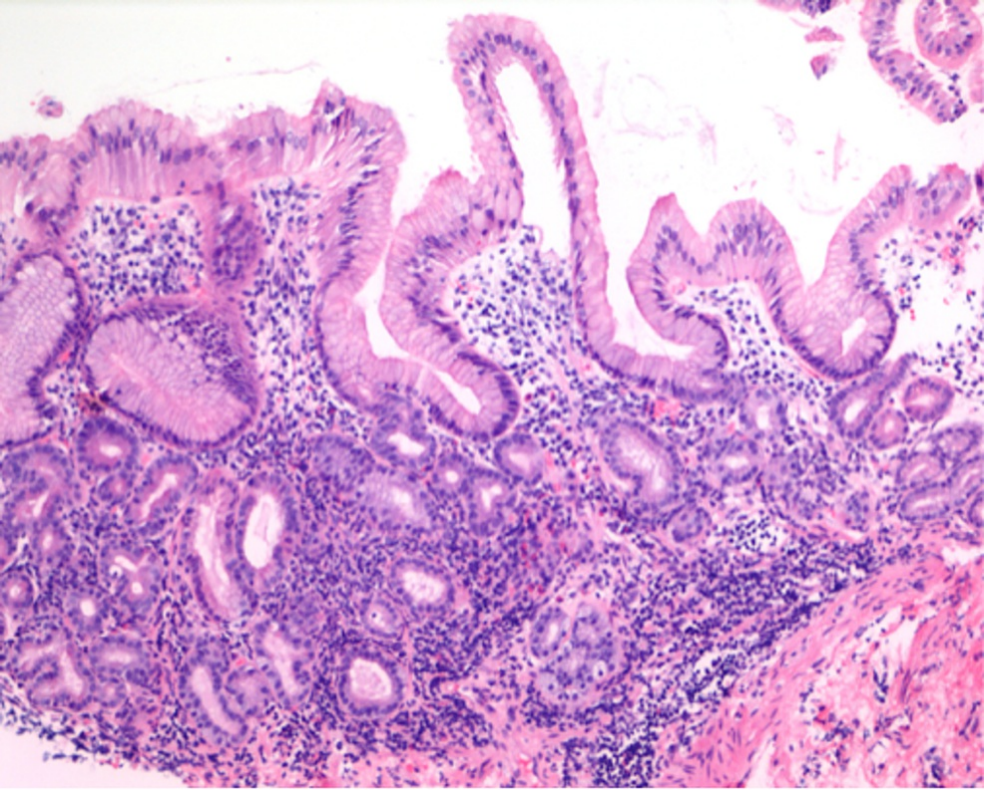
Body of the stomach showing chronic gastritis with intestinal metaplasia of the glands

**Figure 2:**
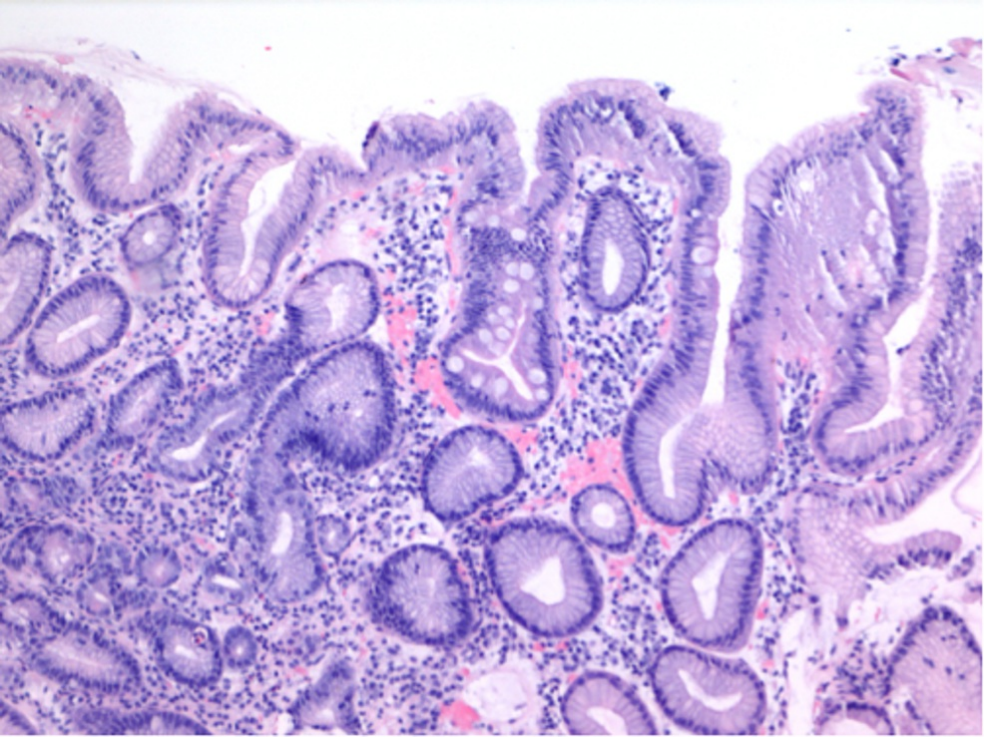
Antrum of the stomach consistent with reactive gastropathy

We proceeded with endoscopic evaluation for further evaluation. Esophagogastroduodenoscopy (EGD) showed diffusely atrophic mucosa in the entire stomach without erythema or ulceration. Biopsies from the gastric body showed chronic gastritis (Figure 1) and patchy areas of metaplasia without dysplasia in the antrum (Figure 2). *Helicobacter pylori* testing on the pathology specimen returned negative. Colonoscopy was significant for two non-bleeding cecal angioectasia and a 2-mm tubular adenoma in the proximal ascending colon. Colonic angioectasia were treated empirically with argon plasma coagulation. Celiac serology was unremarkable. Additional laboratory studies returned positive for parietal cell antibody (PCA) and intrinsic factor blocking antibody (AIF), confirming the diagnosis of PA. She was subsequently advised to initiate oral iron and vitamin B12 supplementation, which led to normalization of hemoglobin from 8.1 g/dL to 11.8 g/dL (reference range: 11.7-15.5 g/dL).

## Discussion

AIH is known to be associated with several different autoimmune diseases. Approximately 20%-30% of patients with type 2 AIH have been reported to have additional autoimmune diseases [3], suggesting a genetic predilection that is well established in the medical literature. Advances in medical and immunological sciences have made it easier to detect patients with AIH. AIF antibody (Ab) has a sensitivity of 37% and specificity of 100%, while PCA Ab has a sensitivity of 81.5% and specificity of 90.3%. The combination of the two antibodies for PA has 73% sensitivity and 100% specificity [4]. To the best of our knowledge, there is only one documented case of AIH co-occurring with PA in the medical literature (Table 1).

**Table 1:** Only known documented case of AIH co-occurring with PA in correlation to our case PCA, parietal cell antibody; AIF, intrinsic factor blocking antibody; AIH, autoimmune hepatitis; PA, pernicious anemia

Study	Age (years)	Gender	Coexisting autoimmune diseases	Comorbidities	Positive antibodies besides PCA and AIF
De Block et al., 2000 [5]	45	Male	Type 2 AIH, type 1 diabetes, pernicious anemia	Gastric carcinoid tumor	Anti-nuclear, Anti-actin, anti-mitochondrial antibodies
	70	Female	Type 1 AIH, hypothyroidism	Takotsubo cardiomyopathy	Anti-smooth muscle antibody, anti-nuclear antibody

A retrospective study by Teufel et al. examined a cohort of 278 patients with AIH for concurrent autoimmune diseases. Out of 278 patients, 111 (40%) were found to have co-occurring autoimmune diseases, including one patient with AIH and autoimmune gastritis. None of the patients were found to have PA. The most common coexisting autoimmune disease was autoimmune thyroiditis, which was found in 28 (10%) patients. Additional autoimmune diseases seen in patients with AIH were vitiligo, rheumatoid arthritis, Sjogren's syndrome, ulcerative colitis, conjunctivitis, celiac disease, systemic lupus erythematosus, type l diabetes mellitus, multiple sclerosis, polymyalgia rheumatic, and urticaria. Interestingly, the association of other autoimmune diseases in patients with AIH was not significantly correlated with a more severe course or increased recurrence of disease [2].

Although literature review revealed that the co-occurrence of AIH and PA was rare, its co-occurrence with other autoimmune diseases was frequent. In a retrospective study by Zulfiqar and Andres examining the association between PA and polyendocrinopathy, 188 patients with PA were followed between 2000 and 2010. Out of the 188 patients, only one female patient was found to have both to have AIH and PA [6]. Similarly, in a mini-review by Shizuma on concomitant cases of PA and autoimmune liver diseases, only one single case of AIH and concurrent PA was identified [3]. Both diseases have a high predilection for autoimmune thyroiditis and female predominance [6]. No specific HLA (human leukocyte antigen) type was associated with either disease [7,8]. Further studies are needed to delineate the genetic association between the two diseases.

In regard to the single known case in the medical literature referenced in Table 1, the subsequent development of gastric carcinoid tumor was a unique feature. Although serology was positive, as in our patient, for PCA and AIF [5], laboratory studies were also positive for anti-liver/kidney microsomal antibodies (LKM), consistent with the diagnosis of type 2 AIH. In their patient, the diagnosis of carcinoid tumor came 16 years after his diagnosis of AIH. Although our patient did not have features suspicious for carcinoid tumor, it potentially increases her risk of developing it in the future, as studies have shown an increased prevalence of carcinoid tumors in patients with PA [9,10]. It is vital that the clinician identifies this association as it can potentially allow patients with AIH and PA to get screened for gastric carcinoid tumors and have it treated at an earlier stage. Testing serum gastrin levels in such patients may allow early detection and subsequent treatment. It may also prevent patients from developing complications of carcinoid tumors, such as local mechanical effects or neuroendocrine symptoms [11]. As evident by our patient's history and the literature mentioned above, it is essential to consider PA as a differential for patients presenting with AIH and anemia. It is also vital to consider screening these patients for other autoimmune disorders, as the prevalence and understanding of polyautoimmunity continue to rise.

## Conclusions

The co-occurrence of AIH and PA is extremely rare. In addition to our case, there is only one documented case of AIH associated with PA. We presented a case of a 70-year-old woman with AIH who presented with anemia and was subsequently found to have PA. This highlights the importance of keeping pernicious anemia as a deferential for similar patients presenting with AIH and anemia. Furthermore, there may be value in screening such patients for carcinoid tumor given the increased prevalence of carcinoid tumor in patients with PA.
